# A novel polyepitope vaccine elicited HIV peptide specific CD4+ T cell responses in HLA-A2/DRB1 transgenic mice

**DOI:** 10.1371/journal.pone.0184207

**Published:** 2017-09-01

**Authors:** Haitao Liu, Wei Shen, Jiayi Shu, Zhihua Kou, Xia Jin

**Affiliations:** 1 Viral Disease and Vaccine Translational Research Unit, Institut Pasteur of Shanghai, Chinese Academy of Sciences, Shanghai, China; 2 Institute of Molecular Ecology and Evolution, East China Normal University, Shanghai, China; 3 Shanghai Public Health Clinical Center and Institutes of Biomedical Sciences, Key Laboratory of Medical Molecular Virology of Ministry of Education/Health, Fudan University, Shanghai, China; 4 State Key Laboratory of Pathogen and Biosecurity, Beijing Institute of Microbiology and Epidemiology, Beijing, China; Rockefeller University, UNITED STATES

## Abstract

Human immunodeficiency (HIV) infection is a leading global health problem that causes approximately one million deaths each year. Although antiretroviral therapy can slow down the disease progression and improve the quality of life of infected individuals, it cannot eradicate the virus. A successful vaccine is one of the most cost-effective alternatives to control the incidence and mortality of HIV infection. CD4+ T cells play a key role in orchestrating other forms of human immune responses, therefore, an HIV vaccine that includes a component capable of eliciting CD4+ T cell responses is highly desirable. To this end, we have previously designed a polypeptide vaccine comprised of multiple CD4+ T cell epitopes. In the current study, we tested the immunogenicity of this vaccine in mouse models by using IFN-γELISPOT and intracellular cytokine staining assays. We found that several epitopes in this vaccine elicited CD4+ T cell immune responses in both congenic mice and human HLA-A2/DRB1 transgenic mice. These new epitopes may be further tested for their ability to augment immune responses elicited by other forms of HIV vaccines.

## Introduction

The latest UNAIDS statistics has showed that there were 36.7 million people living with Human immunodeficiency virus (HIV) infection in 2015[1]. A proportion of these infected individuals will have acquired immune deficiency syndrome (AIDS) and die in the absence of virus eradication. Although antiretroviral therapy (ART) is effective at slowing down the progression to AIDS, its global coverage only reached 46% by the end of 2015[1]. In addition, ART cannot completely clear HIV from infected patients. Recently, “treatment as prevention” has been demonstrated to be rather effective at preventing new infection in selected high-risk groups[2], however, a prophylactic HIV vaccine is less expensive and more suitable for use in a larger population. Thus, the development of an efficacious HIV vaccine is a high priority research area.

Protection against HIV infection needs the orchestrated effort of innate, humoral and cellular immune responses locally as well as systemically. Thus, an efficacious HIV vaccine requires the elicitation of these immune responses. However, to accomplish this task, HIV vaccine research has went through a tumultuous process. Large HIV vaccine clinical trials that were based on the stimulation of CD8+ T cell response alone (Step (HVTN 502) and Phambili (HVTN 503), Phase IIb), and those that activated primarily type-specific antibody responses (Vax003 and Vax004, Phase III) have failed to confer protection against HIV infection[3]. Only in RV144 phase III clinical trial using prime with ALVAC vectored vaccine, following by boost with recombinant HIV envelope protein, a 31% protective efficacy was demonstrated[4]. Surprisingly, V1V2 specific IgG non-neutralizing antibody response was correlated with protection[5], and a robust CD4+ T cell response, as demonstrated by the production IL-2, IL-4, IFN-γ, and TNF-α from antigen-specific T cells, was associated with a significantly decreased risk of HIV acquisition[6]. Other than difficulty in ascertain the specific attributes of protective immunity against HIV acquisition, the design of an HIV vaccine needs to consider the extreme genetic diversity of HIV quasispecies, so that one efficacious vaccine can afford protection against numerous viral variants.

Although active CD4+ T cells are more susceptible to HIV infection, and the depletion of CD4+ T cells is one of the hallmark of HIV pathogenesis[7, 8], because its central role in regulating antibody and CD8+ T cell responses[9, 10], an efficacious HIV vaccine requires the induction of adequate CD4+ T cell response. Previously, utilizing bioinformatics methods, we have customer designed a polypeptide HIV vaccine comprised of 20 CD4+ T cell epitopes based on Chinese HLA-DR types and the prevalent HIV genotypes in China. This vaccine, in theory, could elicit HIV-specific CD4+ T cell responses in 98.1% of Chinese[11]. We then expressed the polyepitope antigen as a recombinant protein and purified it to high degree of purity[12]. In the current study, we further tested the immunogenicity of this vaccine candidate in mouse models. Our results showed that this experimental vaccine induced antigen specific CD4+ T cell immune responses, and some epitopes were more immunogenic than others. Such results may inform the design of a more immunogenic polypeptide vaccine against HIV.

## Materials and methods

### Vaccine construction and protein purification

A recombinant protein vaccine comprised of multiple epitopes was constructed as we have described[12]. Briefly, genes encoding the 20 predicted CD4+ T cell epitopes (Th20), with gene encoding a GPGPG spacer between epitopes, were codon optimized for expression in *E*. *coli* and then synthesized and cloned into a pET30+ plasmid, and used to transform *E*. *Coli* BL21 (DE3) (Genescript, Suzhou). The positive colonies of transformed *E*. *Coli* were selected and cultivated in Luria-Bertani (LB) medium (tryptone 10 g/L, yeast extract 5 g/L, NaCl 10 g/L) supplemented with 50μg/ml kanamycin at 37°C and 180 rpm. When the OD_600_ value reached 0.6–0.8, the cells were induced with 1mM isopropyl-β-D-thiogalactopyranoside (IPTG) for 5h at 37°C. The culture was harvested and centrifuged at 10,000*g* for 10min at 4°C. The cell pellet was resuspended in chilled Native Lysis Buffer containing 1mMphenylmethylsulfonyl fluoride (PMSF) before being lysed by an ultrasonic cell disrupter. After three cycles, the lysed pellets were mixed with an equal volume of Native Purification Buffer at pH 8.0, mixed for 1h at 4°C and centrifuged for 60 min at 10,000 rpm at 4°C. The inclusion bodies were collected, dissolved in 10ml of Guanidinium Lysis Buffer (pH7.8) and centrifuged at 15,000 rpm for 60 min at 4°C. Next, 40ml Denaturing Binding Buffer and 2ml Ni-NTA Agarose were added into the supernatant and mixed at room temperature for 3h. The lysate solution was passed through a prepared Purification Column containing settled resin. The column was washed once with Denaturing Wash Buffer (pH 6.0) and then the target protein was eluted by adding Denaturing Elution Buffer (pH 4.0). Protein elutions were collected and protein was processed for sodium dodecyl sulfate polyacrylamide gel electrophoresis (SDS-PAGE) and Western blot analyses. The target protein was concentrated with 3kD centrifugal filter tubes (Millipore, USA). The concentration was determined by BCA protein assay kit (Thermo Fisher, USA). The purified polypeptide was stored at -80°C until use.

### Experimental animals

BALB/c X C57BL/6 F1 (CB6F1) mice were bred under specific pathogen free (SPF) conditions and standardized diets were given at the animal facilities (Institut Pasteur of Shanghai, China). Human HLA-A2/DRB1 transgenic mice were created from the C57BL/6 mouse strain by using transgenic and gene knockout technology to express only human HLA-DRB1 and HLA-A2.1, but not H-2 K^b^/D^b^ or H-2 IA^b^ [13] (kindly provided by Dr. Yusen Zhou, Beijing Institute of Microbiology and Epidemiology, Academy of Military Medical Sciences, Beijing, China). All experiments were carried out in SPF animal facilities at either Institut Pasteur of Shanghai, or Beijing Institute of Microbiology and Epidemiology. All animal experimental protocols were approved by Institutional Animal Care and Use Committees at both institutions, followed the ARRIVE Guidelines Checklist (S1 ARRIVE Guidelines Checklist).

### Immunization

Groups of randomly selected 3–6 CB6F1 or HLA-A2/DRB1 transgenic mice (6–8 weeks of age) were immunized intramuscularly (i.m.) with either 10μg Th20 protein vaccine diluted into 50μl PBS and mixed with 50μl of 2% Alhydrogel adjuvant (InvivoGen, USA), or 50μl PBS plus an equal volume of Alhydrogel as control. Immunization was performed three times with two-week intervals. CB6F1 and transgenic mice groups were sacrificed by cervical dislocation at 1 and 2 weeks after the final immunization, respectively; spleens were collected and ground individually into single cells with a 70μm cell strainer, and the harvested cells were washed and then used for ELISpot or ICS assays. No spleen samples were pooled for assay usage.

### ELISpot

The ELISpot assay was performed using our published protocol for human T cells [14], with minor modifications for use in mice. Briefly, antigen specific T cell responses were determined by IFN-γ enzyme-linked immunospot (ELISpot) assay according to the manufacturer's protocol provided with the mouse IFN-γ ELISpot (HRP) kit (Mabtech). Wells of PVDF membrane-bottomed 96-well plates (Millipore, USA) were coated with 15μg/ml capture antibody AN18, left overnight at 4°C. The next day, the plate was washed 5 times with PBS and then blocked with cell medium (RPMI 1640 with 10% FBS and 1% penicillin/streptomycin) (Thermo Fisher Scientific) at room temperature for 1 h. After washing once with medium, each well was supplemented with 3×10^5^ cells harvested from CB6F1 mice or 6×10^5^ cells harvested from HLA-DRB1 mice. Then each individual peptide (10μg/ml) contained in the Th20 vaccine was added to separate wells, using ConA (5μg/ml) as positive control and PBS as negative control. Two replicates were used in CB6F1 mice and three were used in HLA-DRB1 transgenic mice. Plates were incubated for 48h at 37°C in 5% CO_2_. Plates were washed five times with PBS, then incubated with HRP conjugated anti-IFN-γdetection monoclonal antibody (R4-6A2) (1:1000 Dilution) for 1h at RT, and then washed 5 times before Streptavidin-HRP (1:1000 Dilution) was added for an another hour at RT. Plates were washed 5 times, spots were visualized by adding 50μl of TMB substrate (Mabtech), then rinsed with tap water to stop the reaction. The plate was air-dried in the dark. The spots were enumerated with an ImmunoSpot analyzer (CTL, USA).

### Intracellular cytokine staining

The ICS assay was performed using our published protocol for human T cells[14], with minor medications for use in mice. Briefly, the specific phenotype of antigen specific cells was determined using intracellular cytokine staining (ICS) assay. Cells at 2.8×10^6^/well were aliquoted into each well of a 24-well tissue culture plate. Peptide pool (containing Pol892, Nef127, Gag297T, Pol295, Gag297S and Env549, each at the concentration of 10μg/ml) was added to each well, PMA (50ng/ml) & ionomycin (1μg/ml) stimulation was used as positive control, unstimulated cells were used as negative controls. Cells were incubated at 37°C 5% CO_2_for 1 hour, and then 1μg/ml Brefedlin A (GolgiPlug, BD) was added to block cytokine secretion. After incubation at 37°C 5% CO_2_ for another 5 hours, the cells were surface stained with anti-CD3-AF488 (145-2C11, BD biosciences), anti-CD4-BV786 (MR4-5, BD biosciences) and LIVE/DEAD aqua (Life Technologies) for 30 minutes at 4°C; following washing and permeabilization, the cells were stained with an anti-IFN-γ-PE antibody (XMG1.2, Biolengend) for 30 minutes at 4°C. Cells were washed twice with PBS containing 0.5% FBS, and then resuspended in 200μl 4% paraformaldehyde (PFA). The stained cells were analyzed by a Fortessa flow cytometer (BD biosciences) and then by FlowJo software.

### Statistical analysis

Data were presented as mean ± standard deviation (SD). Differences among and between groups were determined by Mann-Whitney test and student t-test, respectively. A p<0.05 value was considered significantly different statistically. Positive peptides in the ELISpot assays were determined by Mann-Whitney test among groups, using control as a comparator. In CB6F1 mice, background was very low, most of the well had no spots, and no wells has more than two spots in the control group. Thus, we added one more inclusion criterion: Only peptides that induced more than 50 spots/million splenocytes would be considered as a positive peptide.

## Results

### Th20 epitope protein expression and purification

**Fig 1:**
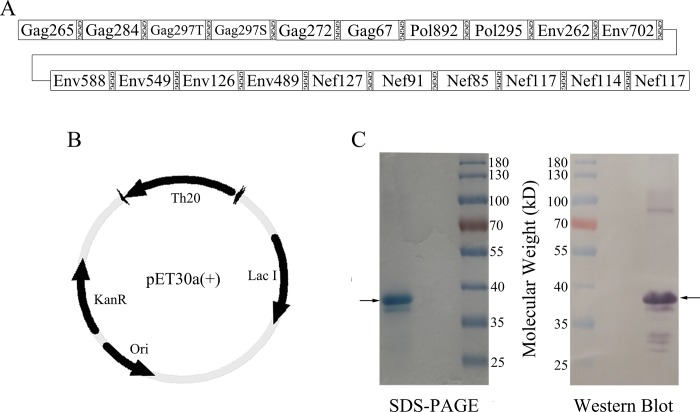
Immunogen design and production. (A) Epitopes were linked by GPGPG spacers to form a single polypeptide antigen. Names of these epitopes were showed in boxes. (B) Genes encoding the Th20 epitopes and spacers were synthesized and cloned into pET30a(+) plasmid and then expressed in a E.coli protein expression system. (C) The expressed Th20 protein was analyzed by SDS-PAGE and Western Blot. Th20 epitopes protein (8μg) was separated by SDS-PAGE on a 10% gel and stained with Coomassie Blue. An equal amount of protein was analyzed by Western Blot using rabbit anti-His antibody to identify the target protein. Arrows indicate the target protein.

We have previously predicted 20 individual CD4+ T cell epitopes (Shu, 2014), genes encoding these epitopes were joined with genes encoding a GPGPG linker between each epitope and cloned into a pET30a+ plasmid and then expressed as a single polypeptide using an *E*.*coli* protein expression system (Fig 1A and 1B). The final protein product, herein named Th20, is approximated 40kD in size on SDS PAGE, and carries a his-tag to facilitate purification as detected by an anti-His antibody on Western blot (Fig 1C).

### Induction of T cell immune responses in CB6F1 mice

**Fig 2:**
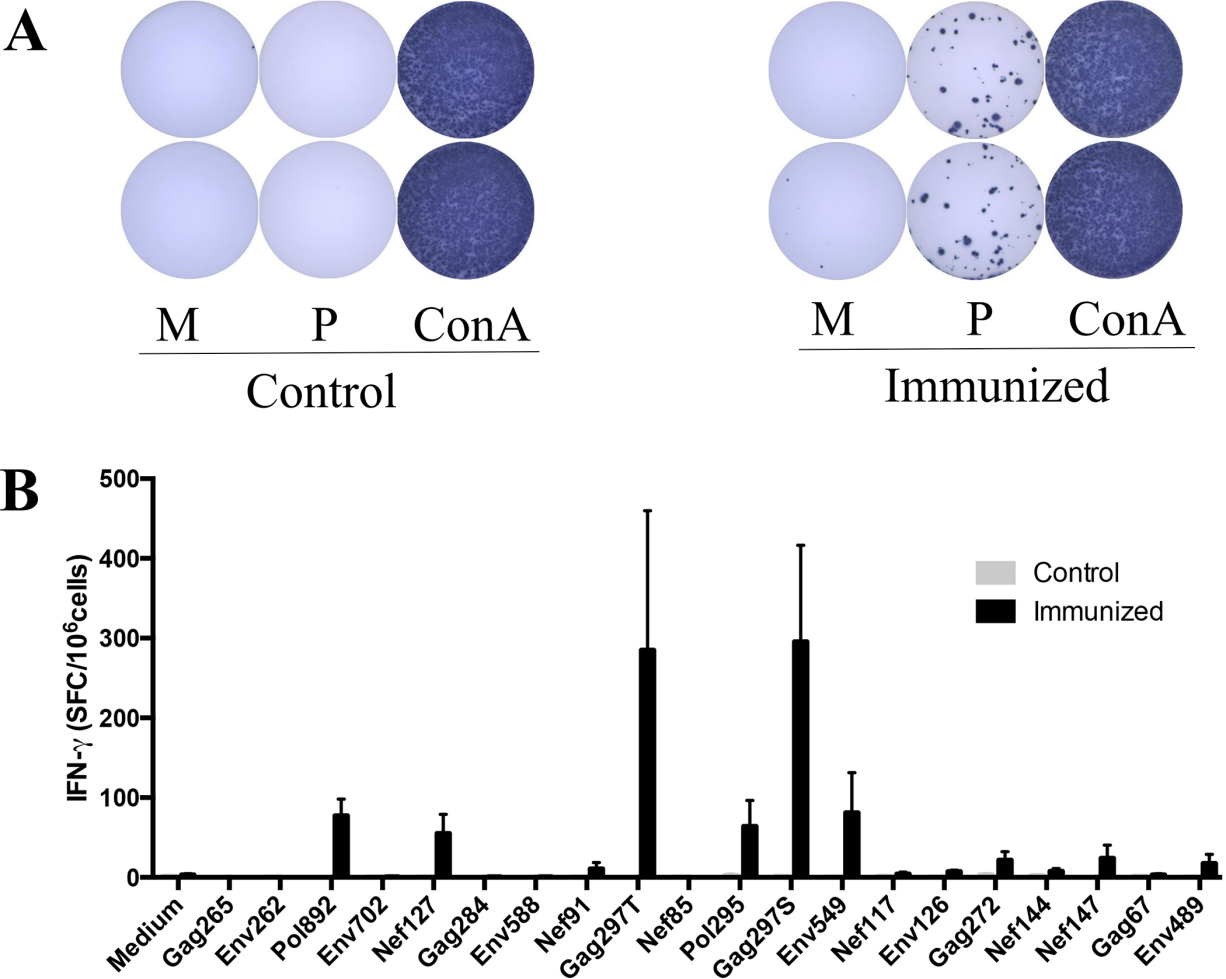
Detection of vaccine-induced immune responses in CB6F1 mice by IFN-γ ELISpot assay. (A) Representative results showing stimulation of spleen cells harvested from immunized mice with negative control medium only, or HIV peptide, or positive control ConA in duplicates. (B) Summary of results from a representative experiment. Each HIV peptide used was labeled by the protein name and the location of the epitope. M = Medium; P = Peptide.

We aimed at examining CD4+ T cell responses elicited by polypeptide vaccine in humanized DR1 transgenic mice to prepare for its immunogenicity testing in humans. To set up the experimental system before moving into the more expensive human DR1-transgenic mice, we first performed the first set of experiments in CB6F1 mice. The immunogenicity of this polypeptide vaccine was first examined in mice with IFN-γ ELISpot assay. Female CB6F1 mice (BALB/c×C57BL/6 F1) at the age of 6–8 weeks were divided into control (n = 3) and immunized (n = 5) groups, and then received 3 inoculations at two weeks interval. One week after the last immunization, mice were sacrificed, spleen cells were extracted, stimulated with individual HIV peptide or negative and positive controls, and then measured for IFN-γsecretion. Fig 2A shows representative control and test wells in which negative controls had few spots and positive controls contained too many spots to count, whereas test wells showed clearly countable number of spots. Overall, six out of 20 HIV peptides, Pol892, Nef127, Gag297T, Pol295, Gag297S, and Env549, induced significantly higher levels of IFN-γ responses in immunized mice than controls (Fig 2B). Here, all control groups had 3–4 mice and immunized group had 5 mice. Results presented are representative of 4 similar experiments.

### Elicitation of T cell immune responses in HLA A2/DRB1 transgenic mice

**Fig 3:**
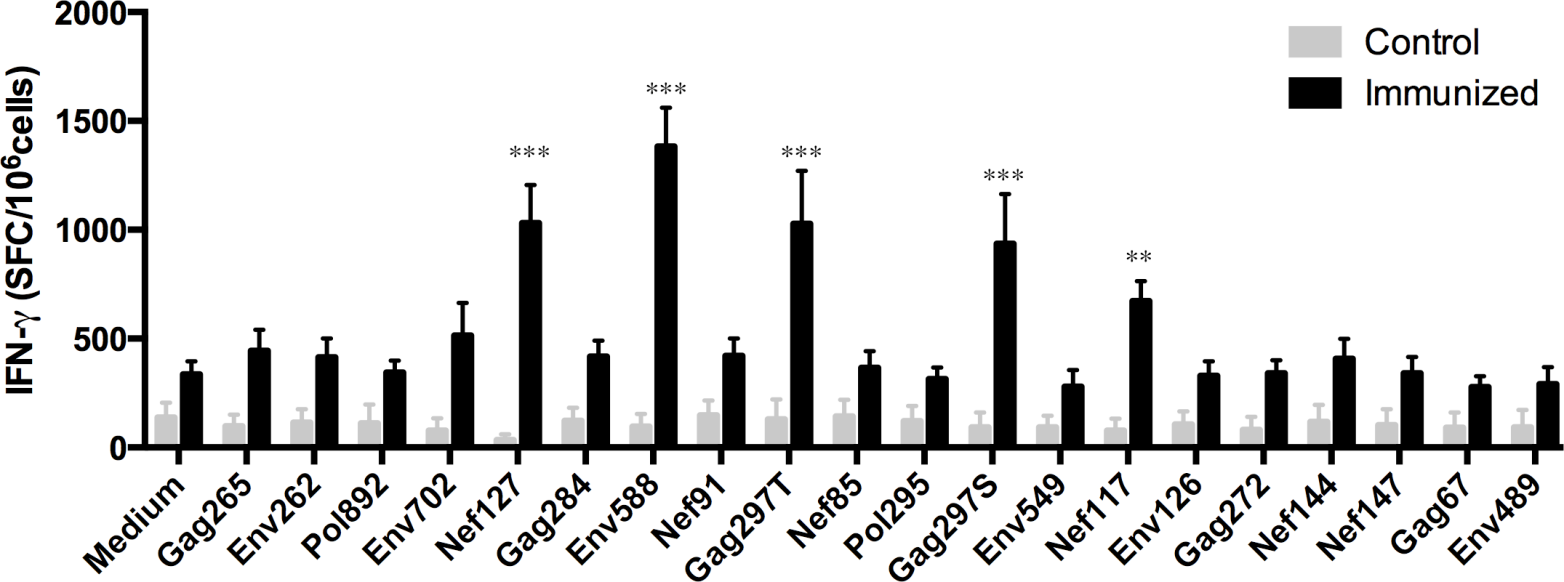
Detection of vaccine-induced immune responses in HLA-DRB1 transgenic mice by IFN-γ ELISpot assay. Female HLA A2/DRB1 transgenic mice at the age of 6–8 weeks were divided into control (n = 4) and immunized (n = 6) groups. Results show the average number of spots per million splenocytes. **: P value <0.01; ***: P value <0.001.

Having demonstrated that the polypeptide vaccine had immunogenicity in CB6F1 mice, we next examined whether the same epitopes contained in the vaccine can be presented by human HLA and generate antigen-specific T cell responses. To do this, we used human HLA-A2/DRB1 transgenic mice which is one of the few mouse strains that express human class II molecules HLA-DR, but not mouse class II I-A or I-E. In vaccinated transgenic mice, 5 out of the 20 epitopes, Nef127, Env588, Gag297T, Gag297S, and Nef117, induced significantly higher levels of T cell responses than controls as detected by the IFN-γ ELISpot assay (Fig 3). The magnitude and diversity of the peptides that stimulated T cell responses are similar but not identical to that in CB6F1 mice. In these experiments, control group has 4 mice and immunized group had 6 mice. Results presented are representative of 2 similar experiments.

### CD4+ T cells are the main source of IFN-γproduction

**Fig 4:**
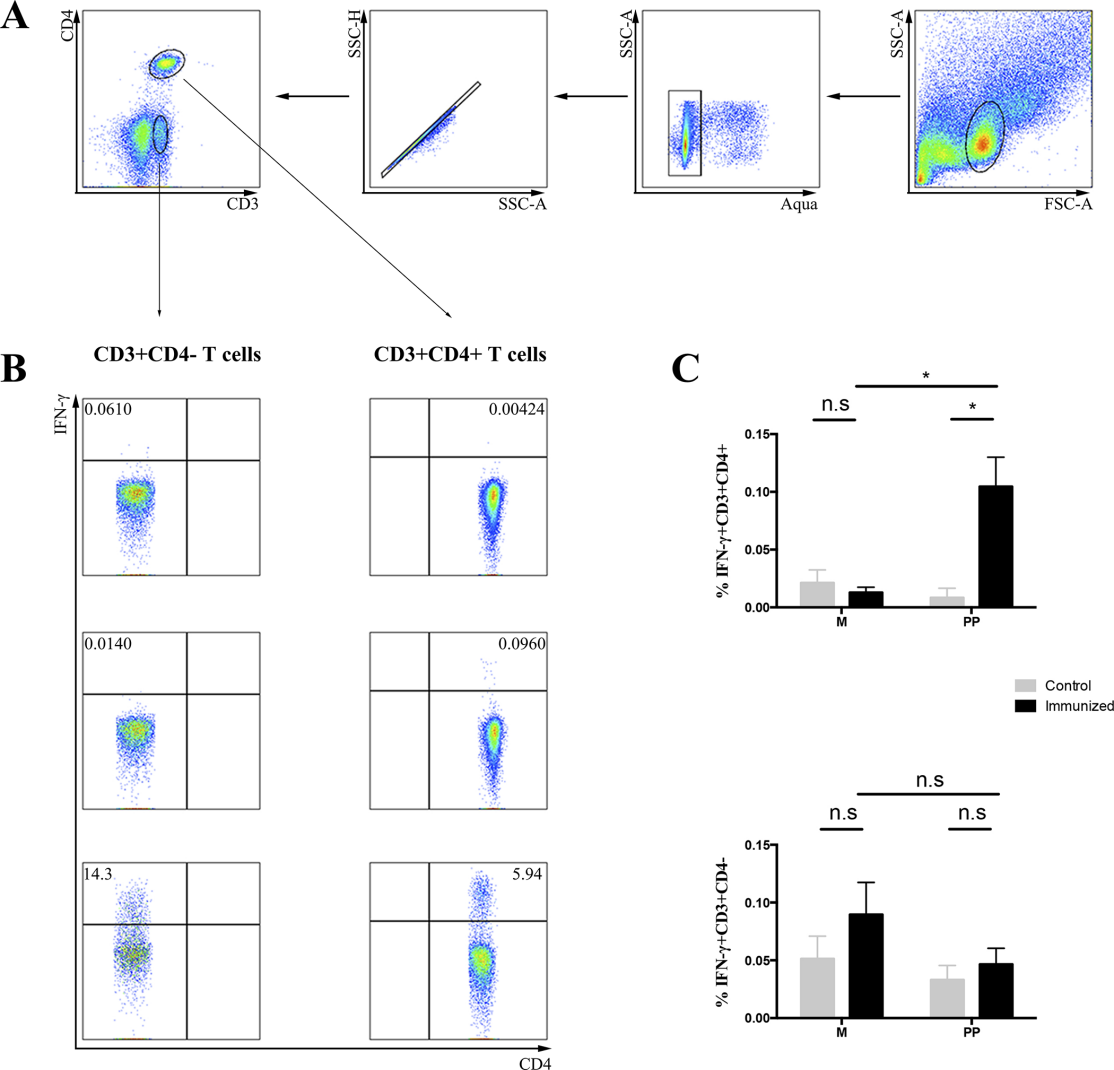
Phenotype analysis of T cell immune responses by intracellular cytokine staining assay. (A) Gating strategy used to identify IFN-γ secretion in CD4+ (CD3+CD4+) and CD8+ (CD3+CD4-) T cell subsets. (B) Representative dot plots of IFN-γsecretion in CD4+ and CD8+ T cells activated by Medium (Top), peptide pool (Middle) and PMA & Ionomycin (Bottom) stimulations. (C) Statistical analysis of IFN-γsecretion in CD3+CD4+ and CD3+CD4- T cells. M = Medium; PP = Peptide Pool. *: P value <0.05; n.s: no significance.

Although IFN-γ ELISpot assays demonstrated that Th20 vaccine elicited T cell responses mediated through murine and human MHC molecules, they do not show the phenotype of responding T cells. To verify the phenotype of T cells that secreted IFN-γ in response to stimulation by HIV peptides, we performed the ICS assay. Following the same gating strategy that allowed comparison of cytokine production between CD4+ T cells (CD3+CD4+) and CD8+ T cells (CD3+CD4-) (Fig 4A and 4B), we assessed the specific subset of antigen specific T cell response. Overall, immunized HLA-DRB1 transgenic mice showed significantly higher percentage of IFN-γproducing CD4+T cells than non-immunized group and peptide non-stimulated group (p<0.05); such differences, however, were not observed for CD8+ T cells (CD3+CD4- group) (Fig 4C). Therefore, in our experimental systems, IFN-γ was predominantly made by vaccine-induced CD4+ T cells. In this experiment, control group had 4 mice and immunized group had 5 mice. No pooled spleen was used

### Epitope affinity analysis

It is interesting to observe that only a proportion of epitopes included in the Th20 vaccine elicited T cell responses, albeit all of them had been predicted to be epitopes. To explore whether peptide-MHC binding affinity *in vitro* affects the immunogenicity of a given peptide *in vivo*, we examined the predicted MHC-II binding affinity of the 20 epitopes included in our Th20 vaccine using an online analysis resource Immune Epitope Database (IEDB). We chose MHC-II binding prediction module in IEDB using the following processes: 1) Choose sequence format: auto detect format; 2) Prediction method: IEDB recommended; 3) Select species/locus: Human, HLA-DR; 4) Select HLA allele reference set: DRB1*01:01; 5) Sort peptides by Percentile Rank; 6) Output format: XHTML table. Based on these processes, we obtained the IC_50_ values (nM) of each of the 20 epitopes.

**Fig 5:**
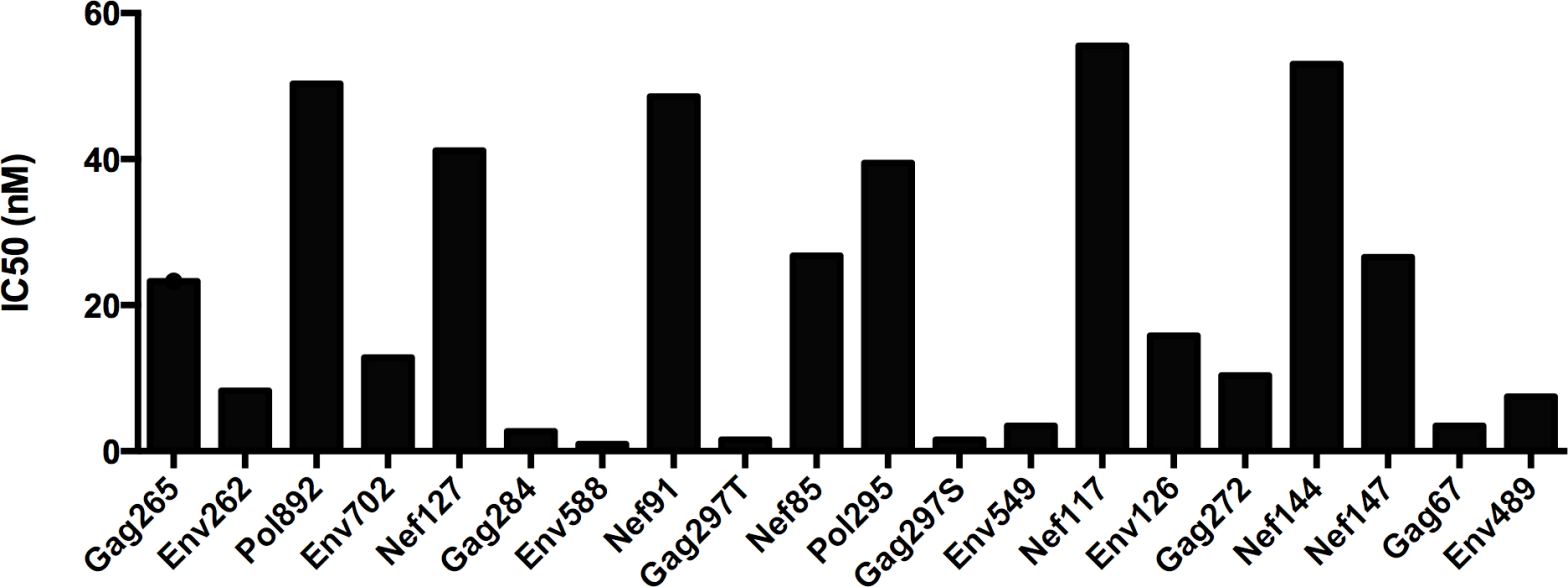
Analysis of epitope affinity and its association with the magnitude of T cell response. The affinity of epitopes included in Th20 epitopes was predicted using IEDB online.

According to IEDB classifications, epitopes with IC_50_ value less than 50nM are considered high affinity, between 50nM and 500nM are considered to be intermediate affinity, and more than 500nM are regarded as low affinity. Based on this classification, the large majority (17/20) of our epitopes are high affinity ones, only three have an intermediate affinity (Pol892: 50.34nM; Nef117: 55.52nM; Nef144: 53.01nM) (Fig 5), and none belongs to low affinity group. When examining the magnitude of immune responses in HLA-A2/DRB1 transgenic mice in comparison to the predicted binding affinities, we found some degrees of correlation, in that high-affinity peptides elicited stronger T cell responses, except for Nef127, Nef117, Gag67. Whether the actual binding affinity, instead of predicted one, associates with precisely an epitope's ability to elicit T cell response *in vivo*, awaits further experimental confirmation.

## Discussion

CD4+ T cells play a key role in orchestrating antibody and CD8+ T cell responses [9, 10]. In a large phase III clinical trial of HIV vaccine, CD4+ T cell responses appeared to be a correlate of protection against the risk of HIV acquisition [4, 6]. Therefore, it is important for an HIV vaccine to induce CD4+ T cell responses. Our current study has demonstrated that a computer-designed vaccine comprised of multiple CD4+ T cell epitopes is capable of eliciting CD4+ T cell responses in mice through either murine or human MHC molecules. Despite not all epitopes stimulated a T cell response, it is reassuring that several epitopes, Nef127, Gag297T and Gag297S elicited specific T immune responses in both congenic mice and human MHC transgenic mice. Thus, the murine models, although not as close to human as monkey model, are a valid tool for proof-of-concept experiments.

While not unexpected, it is discomforting that less than a third of predicted epitopes actually generated a T cell response when administered in the current format as a polyepitope vaccine. One explanation is that epitope binding affinity to MHC dictates its immunogenicity in vivo [15, 16]. Another possibility is that epitopes that stay bound to MHC longer (i.e., having a slower off rate) will have a better chance to engage TCR to stimulate T cell responses [17–19]. A third possibility is that a polyepitope antigen will be processed randomly to yield varying amounts of each of the epitopes, and the most abundance ones will become immunodominant, and demonstrate more immunogenicity [20]. The fourth possibility is that spacer between the epitopes may affect how an epitope is processed and presented.

The GPGPG linkers used in this study were based on previous work in mice and humans [21, 22]. In both studies, a large proportion of the epitopes flanked by the GPGPG linker was adequately presented *in vivo* and stimulated a T cell response. Moreover, the relatively poor epitope presentation we observed in this study might be related to the protein expression systems, a baculovirus expression system was used in previous studies [21, 22], but an E. coli expression system was used in the current studies. Other spacers, such as R/K-R/K motifs (which represent cleavage sites for lysosomal cathepsins B and L) have been applied to join T-helper epitopes in a polyepitope construct [23]. It would be interesting, in future studies, to compare head-to-head between these two type of spacers in epitope-based vaccine design. In any event, which of the above possibility, or a combination of more than one of them, resulted in our current observation is under investigation.

There is little doubt that an effective HIV vaccine should elicit a robust CD4+ T cell response, however, other components of the immune system need to be mobilized at the same time in order to protect against a genetically highly diverse virus such as HIV. To this end, a multivalent vaccine designed with the aid of computational tools, structure biology knowledge, and antibody lineage analyses might represent the future of HIV vaccine development [24].

In summary, we have shown a rationally designed polyepeptide vaccine elicited desired CD4+ T cell responses in both congenic and HLA-DR transgenic mice. Importantly, half of the epitopes recognized by the murine MHC-II were also presented by human HLA-DR in transgenic mice. Therefore, the murine model could serve as a gatekeeper to screen for potential human HIV vaccines, allowing only the most promising ones to move up to the next level of testing. Additionally, understanding the mechanisms that determine immunodominance using mouse model may help to improve the design of a polyepitope vaccine for HIV and other genetically diverse pathogens.

## Supporting information

S1 ARRIVE Guidelines Checklist ARRIVE guidelines checklist. (DOCX)
