# Supplementary material for: A novel polyepitope vaccine elicited HIV peptide specific CD4+ T cell responses in HLA-A2/DRB1 transgenic mice
Source: PLoS One. 2017 Sep 1;12(9):e0184207. doi: 10.1371/journal.pone.0184207 (PMC5580930; doi:10.1371/journal.pone.0184207)

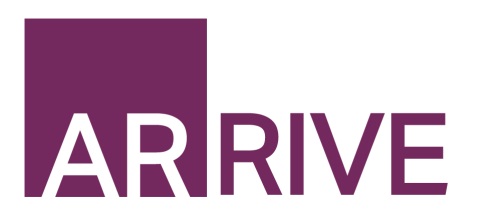


The ARRIVE Guidelines Checklist

A novel polyepitope vaccine elicited HIV peptide specific CD4+ T cell responses in HLA-A2/DRB1 transgenic mice

Haitao Liu^1,2,¶^, Wei Shen^1,¶^, Jiayi Shu^1,4,¶^, Zhihua Kou^3^, and Xia Jin^1,^*

*^1^Viral Disease and Vaccine Translational Research Unit, Institut Pasteur of Shanghai, Chinese Academy of Sciences, Shanghai, China, ^2^Institute of Molecular Ecology and Evolution, East China Normal University, Shanghai, China, ^3^State Key Laboratory of Pathogen and Biosecurity, Beijing Institute of Microbiology and Epidemiology, Beijing, China, ^4^ Shanghai Public Health Clinical Center and Institutes of Biomedical Sciences, Key Laboratory of Medical Molecular Virology of Ministry of Education/Health, Fudan University, Shanghai, China.*

|  | | ITEM | RECOMMENDATION | Section/ Paragraph |
| --- | --- | --- | --- | --- |
| 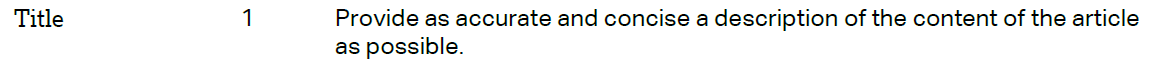 | | | Title |  |
| 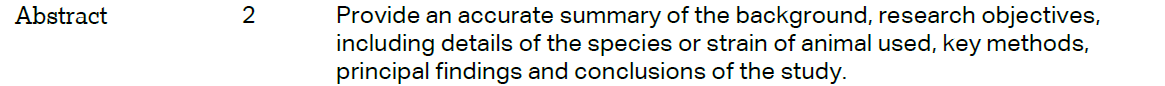 | | | Abstract, Paragraph 1 |  |
| INTRODUCTION | | |  |  |
| 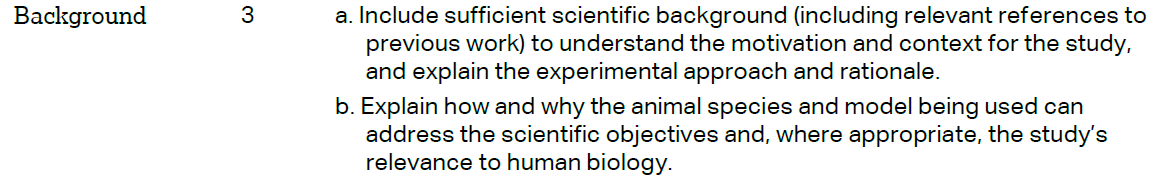 | | | Introduction, Paragraph 2-4 |  |
| 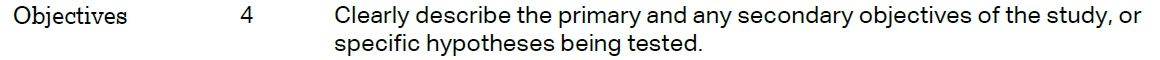 | | | Introduction, Paragraph 4 |  |
| METHODS | | |  |  |
| 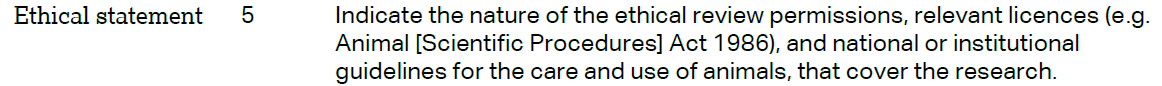 | | | Materials and Methods, Paragraph 6 |  |
| 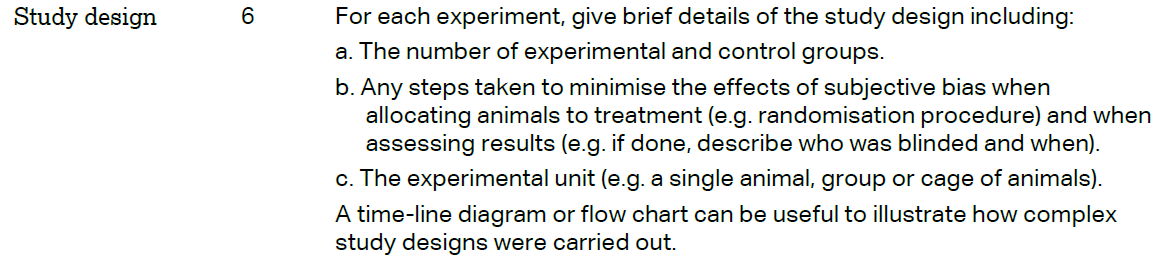 | | | Materials and Methods, Paragraph 7;  Results, Paragraph 12-14 |  |
| 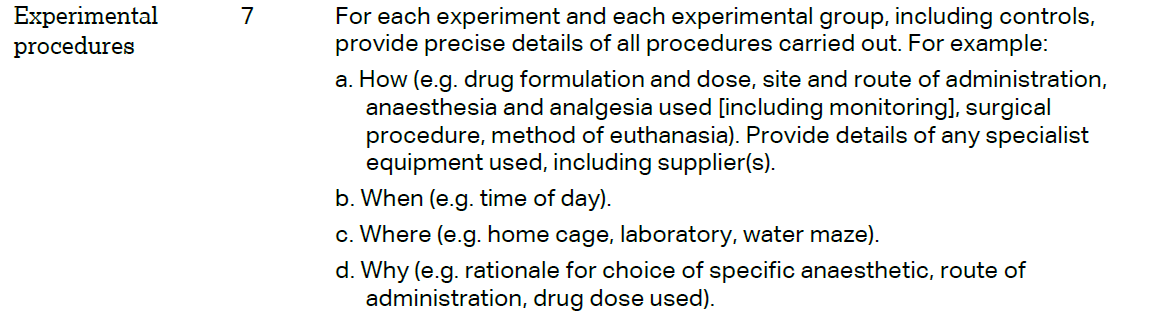 | | | Materials and Methods, Paragraph 7 |  |
| 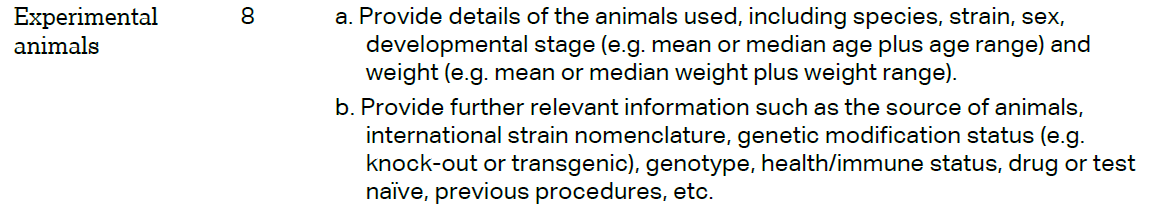 | | | Materials and Methods, Paragraph 6 |  |

The ARRIVE guidelines. Originally published in *PLoS Biology*, June 2010^1^

| 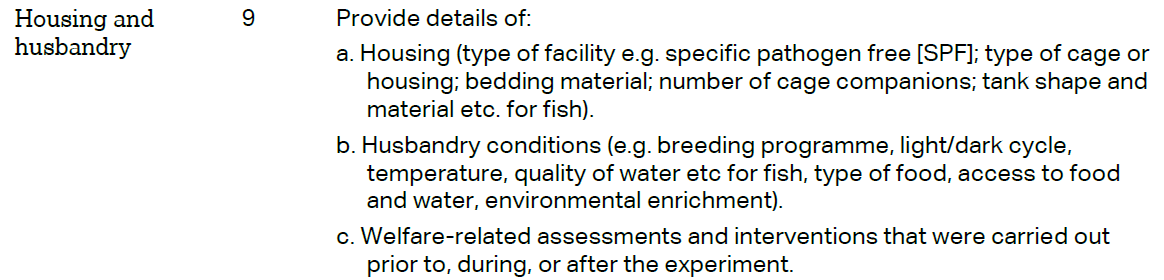 | Materials and Methods, Paragraph 6 | |
| --- | --- | --- |
| 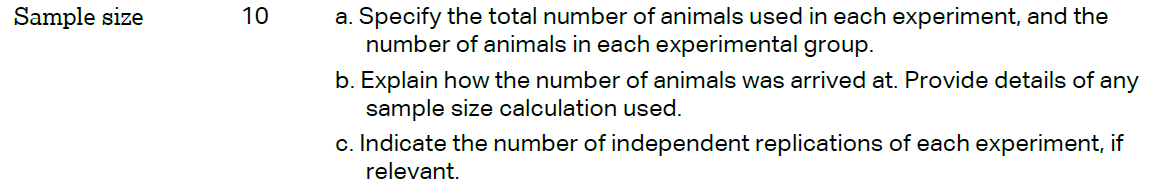 | Materials and Methods, Paragraph 6;  Results, Paragraph 12-14 | |
| 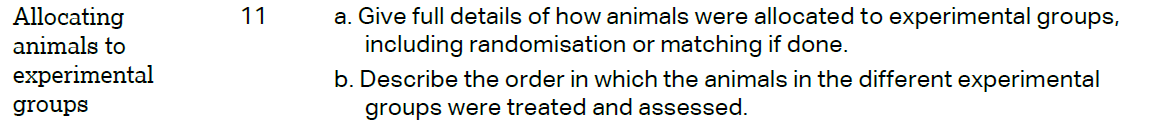 | Materials and Methods, Paragraph 7 | |
| 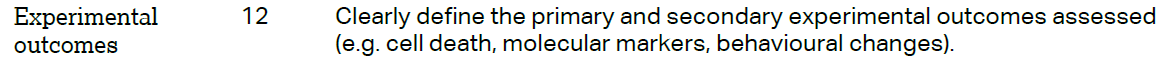 | Results, Paragraph 12-16 | |
| 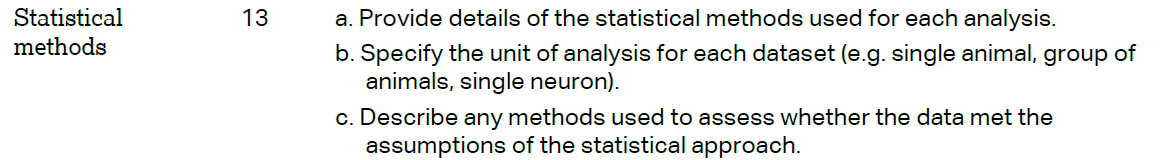 | Materials and Methods, Paragraph 10 | |
| RESULTS |  | |
| 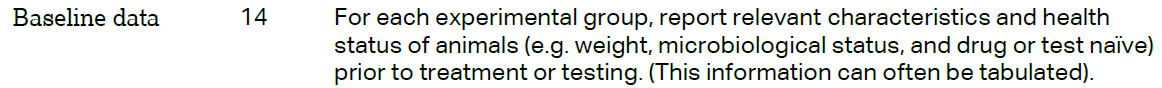 | All mice in each group were health prior to treatment. | |
| 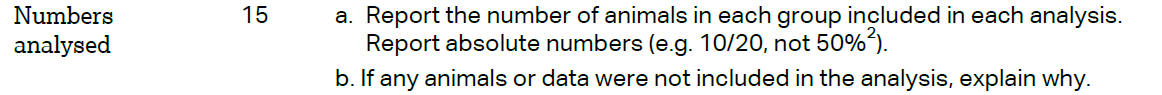 | Results, Paragraph 12-14 | |
| 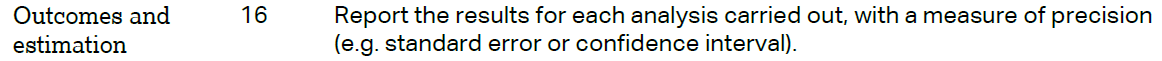 | Results, Paragraph 12-16 | |
| 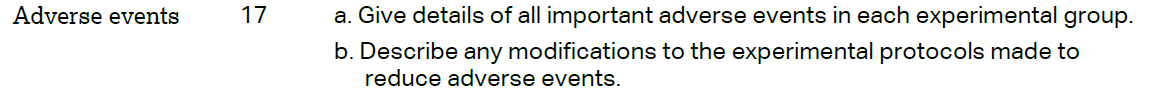 | No adverse events were observed in any experimental group | |
| DISCUSSION |  | |
| 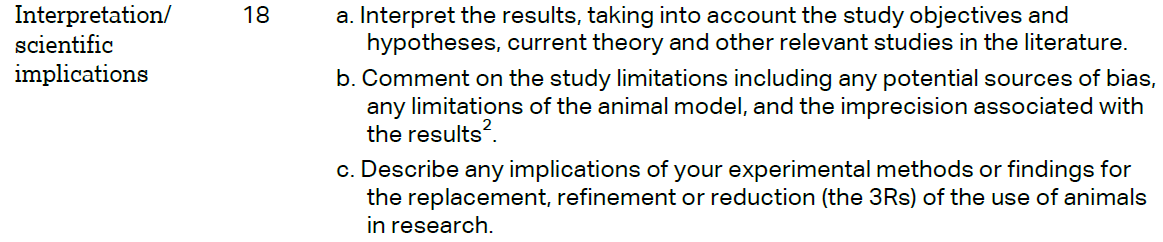 | Discussion,  Paragraph 17-20 | |
| 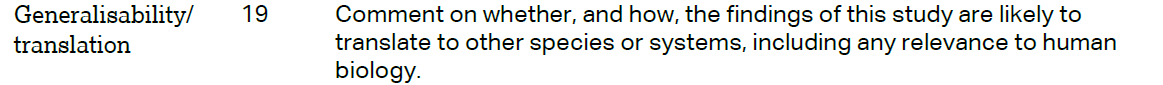 | Discussion,  Paragraph 21 | |
| 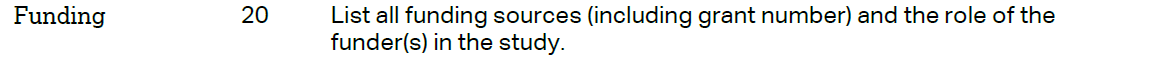 | | Information of Funding only be showed on online system. Namely “The work was funded by the National Major Program Project Grant Against Infectious Diseases (Grant No.2013ZX10001002-002-002).” |


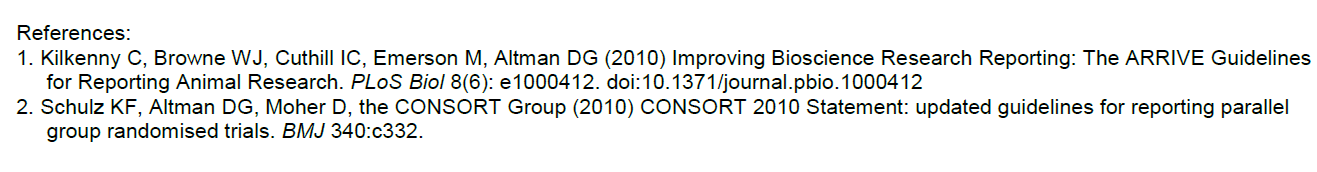

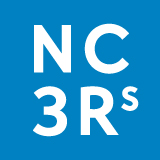

Supplement: S1 ARRIVE Guidelines Checklist — (DOCX) [file pone.0184207.s001.docx]
